# Managing cyclic vomiting syndrome in children: beyond the guidelines

**DOI:** 10.1007/s00431-018-3218-7

**Published:** 2018-08-03

**Authors:** B U.K. Li

**Affiliations:** 1 Children’s Hospital of Wisconsin, Milwaukee, WI USA; 2 Medical College of Wisconsin, Milwaukee, WI USA; 3 Milwaukee, USA

**Keywords:** Cyclic vomiting syndrome, Abdominal migraine, Postural orthostatic tachycardia syndrome

## Abstract

Cyclic vomiting syndrome (CVS) in children is characterized by frequent hospitalizations, multiple comorbidities, and poor quality of life. In the absence of robust data, the treatment of CVS remains largely empiric starting with the 2008 NASPGHAN Consensus Statement recommendations of cyproheptadine for children < 5 years of age and amitriptyline for those ≥ 5 years with propranolol serving as the second-line agent. Comprehensive management begins with lifestyle alterations, and extends to medications, supplements, and stress reduction therapies. Standard drug therapy is organized by the four phases of the illness: (1) *interictal* (preventative medications and mitochondrial supplements), (2) *prodromal* (abortive agents), (3) *vomiting* (fluids/energy substrates, antiemetics, analgesics, and sedatives) and (4) *recovery* (supportive care and nutrition). Because the response to treatment is heterogeneous, clinicians often trial several different preventative strategies including NK1 antagonists, cautious titration of amitriptyline to higher doses, anticonvulsants, Ca^2+^-channel blockers, and other TCA antidepressants. When the child remains refractory to treatment, reconsideration of possible missed diagnoses and further mono- or combination therapy and psychotherapy can be guided by accompanying comorbidities (especially anxiety), specific subphenotype, and when available, genotype. For hospital intervention, IV fluids with 10% dextrose, antiemetics, and analgesics can lessen symptoms while effective sedation in some instances can truncate severe episodes.

*Conclusion*: Although management of CVS remains challenging to the clinician, approaches based upon recent literature and accumulated experience with subgroups of patients has led to improved treatment of the refractory and hospitalized patient.

**Table:** 

**What is Known:** ***•*** *Cyclic vomiting syndrome is a complex disorder that remains challenging to manage.* ***•*** *Previous therapy has been guided by the NASPGHAN Consensus Statement of 2008.*	
**What is New:** ***•*** *New prophylactic approaches include NK1 antagonists and higher dosages of amitriptyline.* ***•*** *Strategies based upon comorbidities and subphenotype are helpful in refractory patients.*

## Introduction

This review addresses the management of cyclic vomiting syndrome (CVS) in children. Described initially in France (1806) and England (1882), the pathophysiology and optimal treatment remain unclear [17]. Significant progress has been made over the last quarter century through formation of national support groups (USA and UK in 1993), published proceedings from two international symposia (1994, 1998), recognition of CVS in adult patients (2006), inclusion in the Rome II–IV classification of pediatric functional gastrointestinal disorders (1999, 2006, 2016), and publication of North American Society for Pediatric Gastroenterology, Hepatology and Nutrition (NASPGHAN) Consensus Statement on CVS (2008) [20]. Passing these milestones has accelerated the number of relevant publications and most importantly led to improved recognition and treatment of CVS.

The pathophysiology appears to involve aberrant brain-gut and cellular pathways including migraine pathways, autonomic and hypothalamic-pituitary-adrenal axis hyperreactivity, and mitochondrial dysfunction. Pharmacotherapy was previously appropriated from that used to treat a spectrum of episodic phenotypes including migraines, seizures, and panic attacks [16]. This paper provides a comprehensive multifaceted treatment approach—lifestyle modification, prophylactic, abortive, and supportive therapy—and discusses further options guided by specific clinical paradigms (by comorbidities, subgroups, and genotype). This review extends the NASPGHAN Consensus Statement by reviewing recent literature and combining it with extensive clinical experience (> 1200 patients) [31].

## What is CVS?

Cyclic vomiting syndrome (CVS) is a functional vomiting disorder characterized by recurring, acute episodes of severe nausea, and vomiting punctuating weeks of baseline health [17]. More than half (58%) require intravenous (IV) hydration in hospital settings and are frequently misdiagnosed with acute gastroenteritis. The differential diagnosis is broad and the NASPGHAN recommendations for diagnostic evaluation are guided by the presence of three alarm symptoms involving the abdomen (severe pain, GI bleeding), metabolism (fasting, high protein triggers), and brain (focal deficits) [20]. Laboratory, radiographic, and endoscopic results are usually unrevealing in 90% and may lead to a diagnosis of psychogenic vomiting.

This disorder affects girls more than boys (55:45), typically begins just prior to primary school, can resolve during adolescence although some will persist into adulthood, and most are predicted to develop migraines. CVS is increasingly recognized in adults, beginning either in childhood or in adulthood. Most children are also affected by multiple comorbid complaints, especially anxiety, fatigue or limited stamina, postural orthostatic tachycardia syndrome (POTS), and chronic daily nausea between episodes so-called coalescent CVS [18]. These added symptoms affect the child when well and may evolve into the primary complaint, confound treatment and adversely impact quality of life.

## Multifaceted treatment by phase of the illness

Fleisher organized an overall management approach based upon the *four* phases of the illness including the following: (1) *interictal period* when lifestyle modifications (drink, eat, exercise, sleep), prophylactic medications and supplements are incorporated to prevent future episodes; (2) *prodromal phase* just prior to the onset of vomiting when abortive medications can sometimes terminate episodes; (3) *vomiting phase* when rescue IV fluids, antiemetics, analgesics, and sedatives provide symptomatic relief; and (4) *recovery phase* when supportive care and nutritional rehabilitation aid recuperation [9].

### Interictal or well interval

Lifestyle modifications themselves can have a significant therapeutic effect. The four components include drinking sufficient (at least maintenance) fluids, eating regularly without skipping meals, exercising regularly (as many become deconditioned), and good sleep hygiene. Fleisher found that by establishing a diagnosis, educating the family about the disorder and recommending simple lifestyle changes reduced the frequency of episodes in 70% of patients *without* the use of medications. The identification of a specific trigger that can be avoided (e.g., dietary monosodium glutamate, sleepovers) can also reduce numbers of episodes.

Prophylactic medications are recommended in children with frequent (≥ every 4–6 weeks) or severe (exceeding 2 days or requiring hospitalization) episodes (Tables 1 and 2). The NASPGHAN Consensus Statement recommended cyproheptadine and amitriptyline for children < 5 years and those ≥ 5 years of age, respectively, with propranolol serving as a second-line agent [20]. Nevertheless, responses to therapy are heterogeneous necessitating a series of drug trials. Although amitriptyline, a tricyclic antidepressant, appears to be the most efficacious and widely used agent, side effects (anticholinergic, cardiac, behavioral) are noted in up to half of treated children and limit its use [3]. Adaptation to side effects such as morning drowsiness is aided by gradual titration of doses in 10 mg increments every 1–2 weeks.

**Table 1:** Classes of prophylactic, abortive, and supportive medications

Class	Agent	Goal	Dosing	Key side effect
Antimigraine	Cyproheptadine	Preventative	0.25–0.5 mg/kg/day divided b.i.d or q.hs	Increased appetite, tiredness
	Pizotifen	Preventative	0.25 mg b.i.d.-t.i.d.	Increased appetite, tiredness
	Amitriptyline	Preventative	Titrate to 1.0–1.5 mg/kg/q.hs.	Constipation, sedation, QT prolongation
	Propranolol	Preventative	0.5–1.0 mg/kg/day divided b.i.d. or t.i.d.	Hypotension, fatigue
	Flunarizine	Preventative	5 mg q.d.	Hypotension
	Mirtazapine	Preventative	7.5–15 mg q.h.	Increased appetite, tiredness
	Sumatriptan	Abortive	6 mg nasal during prodrome	Neck/chest burning
Anticonvulsant	Topiramate	Preventative	2 mg/kg/day divided b.i.d.	Cognitive dysfunction
	Phenobarbital	Preventative	2–3 mg/kg/q.hs.	Cognitive dysfunction
	Levitaracetam	Preventative	1000 mg/day in adults	Cognitive dysfunction
	Zonisamide	Preventative	400 mg/day in adults	Cognitive dysfunction
Antiemetics	Ondansetron	Supportive	0.3–0.4 mg/kg/dose ≤ 16 mg q. 6 h	QT prolongation
	Aprepitant	Preventative	Twice weekly: < 40 kg: 40 mg 40–60 kg: 80 mg > 60 kg: 125 mg	Fatigue, diarrhea
		Abortive	30 min before vomiting, day 2 and 3: < 15 kg: 80, 40, 40 mg 15–20 kg: 80, 80, 80 mg > 20 kg: 125/80/80 mg	
Prokinetics	Erythromycin	Preventative	20 mg/kg/day divided q.i.d.	Abdominal cramps
	Metoclopramide	Supportive	0.1 mg/kg/dose q. 6 h	Irritability, dystonic reaction
Sedatives	Diphenhydramine	Supportive	1.25 mg/kg/dose q. 6 h	
	Lorazepam	Supportive	0.05–0.1 mg/kg/dose q. 4–6 h	Respiratory depression
	Chlorpromazine + diphenhydramine	Supportive	0.5–1.0 mg/kg q. 8 h	Dystonic reaction
Analgesics	Ketorolac	Supportive	0.5–1.0 mg/kg/dose ≤ 10 mg q. 8 h	GI bleeding
Supplements	Coenzyme Q10	Preventative	10 mg/kg/day divided b.i.d.	
	l-carnitine	Preventative	50–100 mg/kg/day divided b.i.d.	Diarrhea, fishy odor
	Riboflavin	Preventative	10 mg/kg/day divided b.i.d.

**Table 2:** Management by disease severity

	Therapy: mild disease	Therapy: moderate-severe disease	Therapy: refractory disease
Lifestyle measures	1. Trigger avoidance 2. > Maintenance fluids 3. Exercise 4. Sleep hygiene 5. Stress reduction	Same + 6. Hospital rescue plan—refers to NASPGHAN Statement 2008	Same + identify specific triggers: stress (bullying), physical (chronic sinusitis), toxic (cannabis use) Re-evaluate for organic disorders (abdominal ultrasound for acute hydronephrosis) Consider intensive rehabilitation program
Abortive	1. Sumatriptan nasal/subcutaneous 2. Ondansetron PO/transdermal 3. Aprepitant PO	1. Aprepitant PO	1. Aprepitant PO
Prophylactic	Optional—if poor response to abortive therapy 1. Coenzyme Q10	< 5 years: 1. Cyproheptadine or pizotifen (outside of the USA) + coenzyme Q10 2. Propranolol 3. Aprepitant ≥ 5 years: 1. Amitriptyline + coenzyme Q10 2. Propranolol 3. Aprepitant Options if side effects: aprepitant, topiramate, phenobarbital, valproic acid, levetiracetam, flunarizine, mirtazapine	1. Aprepitant PO 2. Amitriptyline > 1.5 mg/kg (close monitoring of ECG and blood levels) 3. Combination therapy Amitriptyline + propranolol Amitriptyline + topiramate Amitriptyline + aprepitant Erythromycin + propranolol Rescue sedation: 1. Chlorpromazine + diphenhydramine 2. Dexmedetomidine infusion

Mild disease = no emergency visits or hospital admits; < 6 episodes/year and < 24 h duration. Moderate-Severe disease = occasional-frequent emergency visits and/or hospital admits; ≥ 6 episodes/year and ≥ 24 h. Refractory disease = episodes unchanged/worsening on therapy or missing > 4 weeks of school

If standard agents are either ineffective or poorly tolerated, other medications are used including antiemetics such as aprepitant, anticonvulsants, mitochondrial supplements as well as prokinetic agents, other tricyclic or tetracyclic antidepressants, and Ca^2+^-channel blockers [1–3, 5, 8, 10–12, 14, 21, 24, 30]. The most frequently used anticonvulsants include topiramate and phenobarbital [10, 24]. However, both medications can adversely affect cognitive function. Because subtle mitochondrial dysfunction is thought to affect many children, we generally avoid valproate and incorporate adjunctive mitochondrial supplements such as coenzyme Q10, l-carnitine, and riboflavin [3, 21].

For the frontline pediatric consultant, it is important to use those medications (pizotifen is not approved in the USA [25]) with which one is familiar with both dosing and side effect profile.

### Prodromal phase

During the brief warning phase typically lasting 1.5 h, one can intervene with abortive agents to attempt to terminate the episode. This period is generally characterized by irritability, nausea, pallor, and abdominal pain rather than the classic visual aberrations of migraines. Triptans administered via nasal or subcutaneous routes are more effective if administered during the prodrome before the vomiting commences [12]. These agents are more effective when there is a family history of migraines and the episodes are shorter than 24 h. During the warning phase, some children respond to abortive antiemetics (more to NK_1_ than 5HT_3_ antagonists), a few to analgesics when severe abdominal pain segues into vomiting, and a few to anxiolytics (benzodiazepines) when panic anxiety or anticipation (akin to that prior to chemotherapy) are the triggers.

### Emetic phase

Once the vomiting begins, abortive invention generally fails to end the episode which runs its usual course. Rescue therapy is then initiated to restore hydration and attenuate troublesome symptoms of vomiting, unrelenting nausea, abdominal pain, and headache. Specific components include providing fluid and energy, as well as antiemetic, analgesic, and sedative medications. Reducing stimulation in a dark, quiet, private room with minimum vital sign measures can lessen the vomiting [9]. Although adolescents often describe unremitting nausea as more bothersome than vomiting, there are no effective anti-nausea medications.

In the hospital setting, the saline bolus should be infused on top of maintenance 10% dextrose to concomitantly restore losses and provide cellular energy to terminate ketosis that exacerbates nausea [20]. Providing a specific protocol for the patient to present to the emergency department greatly facilitates care. We typically eschew using the oral route due to the repeated purging, and administer IV, rectal or dermal (reformulated) 5HT_3_ antagonists or IV NK_1_ antagonists (phosphorylated prodrug form) antiemetics which can lessen the pace of vomiting. For pain relief, NSAIDs such as IV ketorolac are preferred to narcotic analgesics. Noting that most (72%) episodes end with premonitory sleep, we have found that induced sleep not only provides symptom relief (when asleep) but can occasionally curtail episodes. Short-acting benzodiazepines (lorazepam) or alternatively a combination of chlorpromazine and diphenhydramine are administered to achieve sedation. This regimen is usually scheduled for the first 24 h, then weaned to an as needed basis as the child improves. In extreme cases in which repeated hospitalizations last longer than a week, the general anesthetic dexmedetomidine can be administered by a continuous infusion in the intensive care setting for close monitoring [13]. These sedative agents are thought to reduce neuronal activation of an aberrant feed-forward emetic pathway.

### Recovery phase

Marked by the end of vomiting to the successful retention of food and drink, this period is typically brief (6 h) unlike the recovery from infectious illnesses. Parents typically describe this endpoint as “a light being switched on” as the instant their child’s skin color returns, eyes open, and energy resumes. The child may be able to resume regular food without any graded reintroduction. However, there are notable exceptions who experience lingering symptoms for up to a week that include intractable nausea with inability to eat (sometimes with low-grade post-ingestion vomiting), persistent dizziness with inability to ambulate, and hyperesthesia with allodynia; antiemetics, H_1_ antagonists or anticholinergic agents, and analgesics respectively are of little help. Management of persisting symptoms continues on as necessary basis. If the lack of nutrition exceeds 5 days, temporary nasojejunal feedings or parenteral nutrition can hasten recovery. If refractory episodes cause frequent and prolonged hospitalizations with a consequent loss of IV access, placement of a subcutaneous port may become necessary.

## Treatment by comorbidity, subgrouping, and genetic profile

How does the consultant best manage the complex patient who fails to respond to the standard NASPGHAN Consensus approach above? We suggest several paradigms on which to base additional treatment options: children with significant comorbidities that warrant independent treatment, those in a subgroup that responds to specific therapies, a few with genetic profiles that suggest alternate medications, and those who are refractory to multiple medications (Table 3).

**Table 3:** Treatment by clinical paradigm

By comorbidity	
Anxiety	Cognitive behavioral therapy, anxiolytics
POTS	Above maintenance fluids, supplemental NaCl, propranolol, exercise
Sleep deprivation	Sleep hygiene, melatonin 3–10 mg q.h.
Fatigue/limited stamina	Coenzyme Q10 10 mg/kg divided b.i.d. into 200–300 mg b.i.d.
–	
By subgroup	
Migraine-related	Antimigraine agents including triptans
Sato variant	Amitriptyline, short-acting ACE-inhibitors/β-blockers for acute hypertension
Mitochondrial dysfunction	Amitriptyline + coenzyme Q10 (± l-carnitine, riboflavin)
Catamenial	Low-estrogen birth control pills (90 day) or depo-medroxyprogesterone
By genotype	
RYR2 mutation	Propranolol

### Treatment by comorbidity

Once viewed as simply a repetitive vomiting disorder, CVS appears to be associated with several comorbid symptoms and conditions, 3.1 per child [18]. These contribute to both child and parent-reported significantly lower health-related quality of life (HRQoL) [27].

The most prevalent comorbidity is anxiety that affects 47% of (59% of school-aged) children with CVS [26]. Anxiety can alter the clinical course in several key ways: as stressful (both positive excitement and negative) events or panic anxiety trigger episodes, or, as school avoidance leads to CVS-induced disability. We demonstrated that HRQoL correlated with trait anxiety and coping abilities rather than with medical severity (frequency, duration of episodes) [28]. Accordingly, a medical psychologist is integral to our treatment team and their behavioral intervention can be the therapeutic tipping point. In more severe cases, the addition of anxiolytic agents (e.g., citalopram, sertraline) to cognitive behavioral therapy may be necessary. If school absenteeism fails to respond to a graded school reentry plan, a comprehensive biobehavioral rehabilitation program may become essential for recovery.

POTS (14% in our cohort, 38% of tested adolescents) commonly affects adolescents with CVS [7, 18, 31]. There is evidence of altered autonomic tone at baseline with elevated sympathetic tone and low to normal parasympathetic tone [29]. Chelimsky reported that management of POTS in adolescents with CVS through fluid and salt supplementation, fludrocortisone, and low-dose propranolol reduced the numbers of vomiting episodes [6].

Chronic daily nausea that peaks in the morning so-called *coalescent CVS* (18% in our cohort) affects adolescent patients (31). In the absence of an effective anti-nausea agent, it is difficult to treat. Anecdotally, amitriptyline, auricular vagal stimulation, and doxylamine-pyridoxine aid some patients.

Addressing sleep deprivation or a low-energy state may also improve outcomes. Behavioral sleep hygiene (turning of all electronic devices, regimented bedtime) and melatonin before bedtime to induce sleep onset may reduce the triggering effect of sleep deficit. In children with chronic fatigue or poor aerobic stamina, the use of frequent or longer-lasting energy sources (protein bars) and coenzyme Q10 (10 mg/kg/day) can improve stamina and enable improved participation in school and extracurricular activities [3].

### Treatment by subgroup

In our CVS program, most children could be further categorized into subgroups, especially migraine-associated [19]. In our pediatric cohort of 355 children, other subphenotypes that required different medications include Sato variant with hypertension and excessive HPA axis activation (6%), mitochondrial dysfunction (5%), and catamenial CVS (9%) [4, 22, 31]. Over half could be classified in a migraine and one or two other subgroups.

*Migraine-related* (90% of our cohort) CVS, either by having a family history of migraines (94%) or personal migraines (38%), constituted the majority of patients and had a substantially higher response rate to antimigraine therapy (80 vs. 36%); therefore, beginning with antimigraine therapy makes sense [19]. When standard therapy fails, this group is treated with anticonvulsants such as topiramate and phenobarbital [10, 24]. Abortive triptans are more effective in those with milder episodes lasting less than 24 h.

The *Sato variant* (6% of our cohort) was described in 1980 as CVS with hypertension, extreme lethargy, and laboratory evidence of a hyperresponsive HPA axis including elevated PGE_2_, ACTH (often 5–6 times the upper limits of normal), cortisol, catecholamines and antidiuretic hormone during the early hours of the episode [23]. The latter three mediators contribute to the pathognomonic hypertension and the ADH can induce oliguria and hyponatremia. These episodes are more severe (more vomiting) and prolonged (3–7 days) than the other subtypes. Sato used anticonvulsants (phenytoin or valproic acid) preventatively, whereas our group uses amitriptyline alone or in combination with other anticonvulsants.

The concept of underlying *mitochondrial dysfunction* (5% in our cohort) originates from findings of a strong matrilineal history of migraine, two highly associated mitochondrial single nucleotide polymorphisms (SNPs), and evidence of efficacy of coenzyme Q10 [29]. Although the precise dose of coenzyme Q10 for CVS is unknown, trials in migraine used approximately 10 mg/kg/day [3]. A serum level at the therapeutic target of 2–2.5× the upper limits of normal can be verified. Although a more complete mitochondrial cocktail including l-carnitine (50–75 mg/kg/day) and riboflavin (10 mg/kg/day) may be beneficial, it frequently incurs pill fatigue [3, 21]. It is worth noting that a subset of children treated with these supplements experience “life changing” improvement in their physical stamina after 3–4 months of treatment.

In adolescent girls, *catamenial* CVS (9% of our cohort, 22% of post-menarchal girls) is a hormonally sensitive variant thought to be triggered by the precipitous fall in estrogen just prior to the onset of menses. This subtype generally begins within a day of menses, prior, or post. Low-estrogen birth control pills (90 days continuously) or long-acting injected medroxyprogesterone can prevent episodes.

Two other subgroups are worth mentioning. The first has unusually long predictable (within 2–3 days) cycles 60 days or longer as if hardwired to a calendar. This group coined *long-cycle*, *calendar-timed* CVS (25%) is especially refractory to both preventative and abortive treatment. The second so-called *cannabis hyperemesis syndrome* (CHS) appears may be triggered by long-standing (> 2 years) high-dose daily cannabis use in adolescent boys or young men [22]. In fact, the similar symptomatology including the use of hot water baths to relieve acute symptoms suggests that CHS may be a subvariant of CVS triggered by excessive cannabis use. Effective treatment involves cessation of cannabis use.

### Treatment by genotype

Boles identified two intertwined SNPs—having both 16519C→T and 3010G→A mutations—that carry a high odds ratio of 17 for CVS (and 15 for migraine) in the Caucasian haplotype H [32]. However, genetic susceptibility may also involve heterozygote nuclear mutations that perturb the stress response by affecting ion channels (RYR2, SCN4A), axonal transport (KIF1B) or energy production (TRAP1). In the RYR2 subgroup, we have had anecdotal success using propranolol [15]. These new findings presage the future possibility that genetic profiles will enable us to predict distinct subphenotypes, clinical courses, and specific treatment.

### Treatment of the refractory patient

If the child or adolescent remains refractory to multiple therapies, what other approaches should be considered? The possibility of persisting triggers (e.g., psychological stressors, chronic sinusitis) and comorbid conditions (e.g., anxiety, POTS) or missed underlying disorders (e.g., acute hydronephrosis, nonfixation of small intestine with volvulus, metabolic crises in toddlers) and toxic exposure (e.g., ipecac in toddlers and cannabis in adolescents). Further investigation should include an abdominal ultrasound *during* a subsequent vomiting episode, and when the history is suggestive, a sinus CT scan and a urine toxicology screen.

When the patient fails to respond to standard therapy, inadequate compliance is common in affected adolescents and can be documented by prescription refills and blood levels for amitriptyline. In addition, the response to specific medications in CVS is quite variable and often requires serial medication trials and dose escalation before efficacy is achieved.

Amitriptyline is the single most effective agent but has anticholinergic, arrhythmogenic, or behavioral side effects that require vigilance. Although half of children experience at least one side effect only 19% have to stop it [5]. Amitriptyline is optimally administered by gradual titration from a starting dose of 0.2–0.3 mg/kg/day and increasing by 10 mg/week to allow adaptation to side effects, monitoring for QT prolongation at baseline and higher doses (> 1.5 mg/kg/day), and to determine if blood levels are therapeutic (> 150 ng/mL). Side effects can sometimes be ameliorated by switching to other TCAs (e.g., nortriptyline, doxepin) administered at similar doses. Intolerance to low doses may indicate slow metabolism induced by CYP2D6 and CYP2C19 mutations. Conversely, a subtherapeutic level at high doses (1.5–3.0 mg/kg q.h.) may reflect ultrarapid metabolism and permit further dose increases until levels become therapeutic. In non-responders, additional medications described above or combinations of a TCA plus either propranolol or an anticonvulsant may be effective (Table 1).

For those with prolonged (7–10 days) episodes requiring repeated hospitalization and IV therapy, deep sedation cannot only provide relief from intractable nausea and vomiting but can sometimes shorten episodes. If IV diphenhydramine or lorazepam does not sedate sufficiently, IV chlorpromazine coupled with diphenhydramine may allow the child to sleep and “reboot” the dysfunctional emetic cycle. In exceptional cases, we have successfully used a published anesthetic protocol of IV dexmedetomidine infusion in the pediatric intensive care unit [13].

For those children and adolescents who have become disabled from academic, extracurricular, and social participation, we recommend an intensive in-patient or out-patient biobehavioral rehabilitation program that involves individual, group and family therapy, physical conditioning, scheduled sleep and awakening, homework, and set consequences for missed school and extracurricular attendance. In these cases, there are usually prominent components of anxiety and deconditioning.

## The future

The future remains promising despite the ongoing challenges of management. There are several programs that specialize in both treatment and research of CVS in both children and adults. There is an active international research consortium that began to study nausea and vomiting in 2013. Lastly, there are potentially promising agents that include new and repurposed medications including antimigraine agents (calcitonin gene-related peptide receptor antibody), auricular vagal nerve stimulation, sedatives (ketamine), and yet unexplored agents including topical capsaicin, cannabidiol and corticotrophin antagonists.

## Summary

Cyclic vomiting syndrome in children remains a devastating disorder beset by frequent hospitalizations, multiple comorbidities and poor quality of life. In the absence of controlled outcomes data, the treatment of CVS remains largely empiric beginning with the 2008 NASPGHAN Consensus Statement recommendations. Comprehensive management begins with lifestyle alterations and includes medications, supplements, and stress reduction therapies. Standard drug therapy is organized by the four phases of the illness: (1) *interictal* (preventative medications and mitochondrial supplements), (2) *prodromal* (abortive agents), (3) *vomiting* (fluid/energy, antiemetics, analgesics, and sedatives), and (4) *recovery* (supportive care and nutrition). Because treatment responses are variable, clinicians often have to trial several different medications including cautious titration of amitriptyline to higher doses, NK1 antagonists, anticonvulsants, Ca^2+^-channel blockers, and other TCA antidepressants. When the child remains refractory to treatment, reconsideration of possible missed diagnoses and further mono- or combination therapy and psychotherapy can be guided by accompanying comorbidities (especially anxiety), specific subphenotype, and when available, genotype. For hospital intervention, IV fluids with 10% dextrose, antiemetics, and analgesics can lessen symptoms while effective sedation in some instances can truncate severe episodes.

## Abbreviations

ACTH: Adrenocorticotropin hormone
CHS: Cannabis hyperemesis syndrome
CVS: Cyclic vomiting syndrome
HPA: Hypothalamic-pituitary-adrenal
HRQoL: Health-related quality of life
NASPGHAN: North American Society for Pediatric Gastroenterology, Hepatology and Nutrition
NSAID: Nonsteroidal anti-inflammatory drug
PGE_2_: Prostaglandin E_2_
POTS: Postural orthostatic tachycardia syndrome
SNP: Single nucleotide polymorphism
TCA: Tricyclic antidepressant
